# Isolation of putative stem cells present in human adult olfactory mucosa

**DOI:** 10.1371/journal.pone.0181151

**Published:** 2017-07-18

**Authors:** Tamara Tanos, Alberto Maria Saibene, Carlotta Pipolo, Paolo Battaglia, Giovanni Felisati, Alicia Rubio

**Affiliations:** 1 Istituto Europeo di Oncologia, Milan, Italy; 2 Otolaryngology Unit, ASST Santi Paolo e Carlo, Department of Health Sciences, Università degli Studi di Milano, Milan, Italy; 3 Otolaryngology Unit, Università dell'Insubria, Varese, Italy; University of Alabama at Birmingham, UNITED STATES

## Abstract

The olfactory mucosa (OM) has the unique characteristic of performing an almost continuous and lifelong neurogenesis in response to external injuries, due to the presence of olfactory stem cells that guarantee the maintenance of the olfactory function. The easy accessibility of the OM in humans makes these stem cells feasible candidates for the development of regenerative therapies. In this report we present a detailed characterization of a patient-derived OM, together with a description of cell cultures obtained from the OM. In addition, we present a method for the enrichment and isolation of OM stem cells that might be used for future translational studies dealing with neuronal plasticity, neuro-regeneration or disease modeling.

## Introduction

The olfactory mucosa (OM) is a specialized tissue that represents the sensory system used for smelling. It is a part of the nervous system that is exposed to frequent injuries due to its anatomical location and therefore, requires continuous cell turnover.

The OM consists in the olfactory epithelium (OE) and the lamina propria (LP), which is a layer of connective tissue located beneath the epithelium. The OE is a stratified neuro-epithelium composed of olfactory neurons, supporting cells and basal cells [1]. The axons of the olfactory neurons extend from the OE to the olfactory bulb.

The OM frame neurons display a regenerative ability that is responsible for continuous neurogenesis indispensable for preservation of the sensory function. Several studies suggest that the capability of the olfactory epithelium to regenerate is due to the presence of a hierarchical stem cell lineage [2, 3]; furthermore, it has been shown that neuronal stem cells are located within the basal layer of the OM and that they can generate neurons and all the cell types present in the OE [4]. Finally, stem cells located in the lamina propria seem to possess unique properties that differentiate them from other mesenchymal stem cells [5–7]. In consideration of all these features, the OM tissue might represent an accessible source of stem cells.

In this investigation, we describe cell populations found in the OM of adult patients and those obtained from OM cell cultures. Furthermore, we produced primary cell cultures from human OM biopsies; therefore, we isolated putative OM stem cells that were identified through the retention of the fluorescent dye denominated PKH26.

## Materials and methods

### Clinical samples

The study has been conducted according to the Declaration of Helsinki. Informed written consent was obtained from all subjects taking part in the study. The research was approved by the Ethics Committee of the San Paolo Hospital, Milan, Italy. Tissue specimens were collected under general anesthesia during routinely planned rhinosurgical procedures. Patients demographics and surgical procedures were reported in Table 1. A single biopsy (3–4 mm^2^) from the posterior nasal septum or from the medial wall of the superior turbinate was collected in the region that was the easiest to access. An incision was made with an 11 blade lancet under endoscopic vision with a 0° scope and the tissue was taken with a Hartmann straight ear forceps. Due to the small quantity of material, some biopsies were used to perform histological analyses (specimens designated as “paraffin samples” in Table 1) and others were used to establish a culture (“culture samples” in Table 1). In order to produce an OM cell culture, the biopsy was immediately placed on ice in an OM medium (DMEM/F12 medium with 10% FBS, 2 mM glutamine, 1% amphothericin B and 100 U/ml penicillin/streptomycin) and transferred for processing.

**Table 1:** Information about tissue specimens used.

	Number	Sample	Gender	Age	Procedure	Comorbidities
cultures samples	1	Posterosuperior septum	M	25	septo turbinoplasty	none
	2	Posterosuperior septum	M	56	revision septoplasty	schizofrenia
	3	Posterosuperior septum	F	15	septo turbinoplasty	none
	4	Superior turbinate	M	45	septo turbinoplasty	none
	5	Superior turbinate	M	20	septo turbinoplasty, endoscopic sinus surgery	trisomy 21
	6	normal tissue of the septum (wide middle and posterior part specimen)	M	46	endoscopic sinus surgery	Grade 3 olfactory neuroblastoma
paraffin samples	7	Superior turbinate	M	49	septo turbinoplasty	none
	8	Superior turbinate	M	23	septo turbinoplasty	none
	9	Posterosuperior septum	M	32	septoplasty and endoscopic sinus surgery	none
	10	Posterosuperior septum	M	59	septoplasty and endoscopic sinus surgery	inverted papilloma

### Cell culture

SK-N-BE (2C) cells were grown in RPMI 1640 medium supplemented with 10% FBS, 2 mM glutamine, 1 mM sodium pyruvate, 0.1 mM non-essential amino acids and 100 U/ml penicillin/streptomycin. U87MG cells were cultured in MEM medium supplemented with 10% FBS, 2 mM glutamine, 1 mM sodium pyruvate, 0.1 mM non-essential amino acids and 100 U/ml penicillin/streptomycin. Primary fibroblasts and mammary human epithelial cells were isolated as previously described (Pece, Cell 2010).

In order to prepare an OM culture, each sample was digested in a Dispase II solution (2.4 U/ml) for 1h, 37°C. The OE and lamina were thus separated by performing a soft brushing with a lancet. The remaining lamina was cut into 1 mm^2^ pieces and transferred into Collagenase I solution, 0,25 mg/ml (10 min, 37°C). The tissue was re-suspended in epithelium/dispase 1:10 with PBS and centrifuged at 1500 rpm for 5 min. Red blood cells lysis buffer (Sigma) was added to remove contaminant cells and it was centrifuged again. The pellet was suspended and plated in OM medium. When adherent cells reached confluency, they were passaged using trypsin.

### FACS

Cells were initially treated with trypsin; then FcR (Miltenyi Biotec) in a blocking solution (PBS 0.5% BSA- 2 mM EDTA) was added to the cells for 7 min. Cells were centrifuged at 200g for 5 min, and suspended in blocking solution. Primary antibodies were added for 7 min and after washing the cells were suspended in PBS for FACS sorting analysis using BD influx Cell Sorting model 646500. Primary antibodies used were: anti-CD31-PE Cy7 (clone 1F11, Beckman Coulter), anti-CD45-FITC (clone J.33, Beckman Coulter), anti-Epcam-FITC (clone HEA-125, Miltenyi Biotec) and anti-CD56-PE (clone 51-10C9, BD Pharmingen). The volume of antibody added per 25,000 cells was: 0.25 μl for anti-CD31, 3 μl for anti-CD45, 20 μl for anti-Epcam and 6 μl for anti-CD56.

To sort PKH26 labeled cells, we first calculated the number of PKH26 positive cells per sphere and divided that by the total number of cells in the sphere. Stem cells constituted a maximum of 5% of the total cells. Then, cells were isolated according to their label intensity. After establishing the negative population gate by the unlabeled control samples we created two more gates: one for the intense PKH26 labeled cells (less than 5% of cells) and one for the intermediate PKH26 labeled population.

Purity of sorted populations was verified by flow cytometry.

### Immunocytochemistry

Cells were fixed with 4% paraformaldehyde in PBS for 10 min and were incubated in a blocking solution (PBS containing 5% normal donkey serum and 0.2% Triton X-100) for 30 min. Then, coverslips were incubated overnight at 4°C with primary antibodies as indicated in Table 2. Immunoreactivities were detected with Alexa Fluor 488-conjugated (1:200, Invitrogen), Cy3-conjugated (1:400, Jackson Immunoresearch Laboratories) and Alexa Fluor 647-conjugated (1:200, Invitrogen) secondary antibodies. Nuclei were stained with 4´,6´-diamidino-2-phenylindole (Dapi, 5 min, 0.2 ng/ml in PBS, Sigma) and actin filaments with Phalloidin-TRITC (40 min, 50 μg/ml, Sigma). Images were captured with an Olympus BX63 microscope and a Leica TCS SP5 confocal microscope equipped with a digital camera and fluorescent images were combined using Fiji. The aspect ratio of the nuclei (i.e., the ratio between the longer axis and the shorter axis of the nucleus) was automatically calculated using Fiji.

**Table 2:** Primary antibodies.

Antibody	Company	Reference	Host	Dilution IF	Dilution WB
Anti-CK14	Covance	PRB-155P	Rabbit	1:500	1:2000
Anti-CK5	Abcam	ab53121	Rabbit	1:100	1:500
Anti-CK8	Home made	TROMA-1	Rat	1:200	1:1000
Anti-Epcam	Abcam	ab32392	Rabbit	1:100	1:2500
Anti-FN	Thermo Scientific	MA1-12597	Mouse	1:100	
Anti-FN	Abcam	ab299	Rabbit		1:4000
Anti-Ki67	Thermo Scientific	RM-9106	Rabbit	1:200	
Anti-Nestin	Millipore	MAB5326	Mouse	1:300	
Anti-NSE	Dako	M0873	Mouse	1:300	
Anti-p63	DAKO	M7317	Mouse	1:100	
Anti-p63	Abcam	ab124762	Rabbit		1:1000
Anti-S100	DAKO	Z0311	Rabbit	1:500	
Anti-S100β	Sigma	S2532	Mouse	1:100	
Anti-Synapto	Abcam	ab16659	Rabbit	1:100	
Anti-SMA	Sigma-Aldrich	A5228	Mouse	1:1000	
Anti-Tuj1	Covance	PRB-435P	Rabbit	1:1000	1:5000
Anti-Vimentin	Bioss	bs-0756R	Rabbit	1:300	
Anti-Vinculin	Sigma-Aldrich	WH0007414M1	Mouse		1:5000

### PKH26 staining

OM derived cells were stained with 1:5000 PKH26 dye (Sigma) in PBS for 5 minutes; they were then washed with 10 ml of PBS 1X, centrifuged at 200g, counted and plated at a density of 5000 cells per ml. Cells were maintained in culture for 7 days before proceeding with a second generation of spheres.

### Sphere formation assay

OM cultured cells were grown in suspension in stem cell (SC) medium [8] with 1% methylcellulose. Cells were plated at a density of 5000 cells per ml in poly-HEMA treated cells and allowed to grow for 7 days. After 7 days sphere formation efficiency was calculated as a ratio between the number of spheres obtained and the total number of cells plated and is represented as percentage (X 100).

Methylcellulose was avoided when studying sphere formation in successive generations.

### Protein isolation/Western blot

Cells were homogenized in lysis buffer (50 mM Tris HCl pH8, 150 mM NaCl, 1% NP-40, 0.5% Deoxycholate, 0.1%, sodium dodecyl sulfate (SDS), 5 mM EGTA and proteases inhibitors (Calbiochem). Protein concentration was determined by Bradford reagent. Protein extracts (15–25 μg) were denatured in Laemmli's sample buffer containing β-mercaptoethanol and bromophenol blue, separated by 10% SDS–polyacrylamide gel electrophoresis and transferred to a nitrocellulose membrane. The membranes were blocked 30 min at room temperature with 5% nonfat dry milk in Tris-buffered saline with 0.1% Tween 20 (TBS-T) and incubated in blocking buffer with the antibodies in Table 2 overnight at 4°C. After washing with TBS-T, membranes were incubated for 1 h at room temperature with horseradish peroxidase-conjugated goat antibodies to mouse (1:2000, Dako), rabbit (1:5000, Amersham), or rat (1:1000, Amersham) IgGs in blocking buffer. Peroxidase activity was detected with Western Lightning Plus reagents (Perkin Elmer).

### Histological analyses

Formalin-fixed biopsies were included in paraffin and serially sectioned into 4-μm sections. Sections were deparaffinated with histolemon, hydrated through graded alcohol series and treated to unmask the antigen (1 mM EDTA pH8 with 0.05%Tween 20 for 50 min 95°C). After cooling the slides, the sections were washed in PBS and incubated in a blocking solution (PBS containing 5% normal donkey serum and 0.2% Triton X-100) for 30 min at room temperature. Samples were incubated in blocking buffer with primary antibodies (Table 2) for 1 h at room temperature. After several PBS washes, the sections were incubated for 1 h at room temperature with Alexa Fluor 488-conjugated (1:200, Invitrogen), Cy3-conjugated (1:400, Jackson Immunoresearch Laboratories), Alexa Fluor 647-conjugated (1:200, Invitrogen), secondary antibodies. Nuclei were stained with Dapi (5 min, 0.2 ng/ml in PBS). Images were captured in a Leica TCS SP5 confocal microscope equipped with a digital camera and fluorescent images were combined using Fiji. All the fluorescent images taken from the tissue sections are 2D projections of 9 consecutive confocal planes located 0.4 μm apart. Some sections were stained with hematoxylin/eosin and images were captured using the Aperio image Scope technologies.

### RNA extraction, Affymetrix GeneChip hybridization and statistical analysis

Total RNA was extracted using commercial homogenization (QIAshredder) and purification (RNeasy Mini Kit) reagents (Qiagen). RNA quality was analyzed with the Agilent 2100 Bioanalyzer (Agilent Technologies).

For quantitative real time PCR, RNA was retrotranscribed using iScript Super Mix (Biorad) and Titan HotTaq EvaGreen qPCR mix (BioAtlas) was used. Expression levels were normalized with respect to the β*-ACTIN* expression. Primers for human *CD271* were (5′-3′): F, GGAGAACGTCACGCTGTCCA, R, GCGCCGACATGCTCTGGAG. Primers for human β*-ACTIN* were: F, ACCCCAGCCATGTACGTT, R, GGTGAGGATCTTCATGAGGTAG.

Biotin-labelled cDNA targets were synthesized starting from 150 ng of total RNA. Double stranded cDNA synthesis and related cRNA was performed with Affymetrix GeneChip® 3' IVT Plus Kit. The fragmented and labeled aRNA was synthesized using the same kit. Labelling was performed according to the manufacturer's protocol.

Each eukaryotic GeneChip® probe array contains probe sets for several *B*. *subtilis* genes that are absent in the samples analyzed (*lys*, *phe*, *thr*, and *dap*). This Poly-A RNA Control Kit contains *in vitro* synthesized polyadenylated transcripts for these *B*. *subtilis* genes that are pre-mixed at staggered concentrations to allow GeneChip® probe array users to assess the overall success of the assay. Poly-A RNA Controls final concentration in each target are *lys* 1:100,000, *phe* 1:50,000, *thr* 1:25,000 and *dap* 1:6,667.

Hybridization was performed using the Affymetrix GeneChip® Hybridization, Wash and Stain Kit. It contains mix for target dilution, DMSO at a final concentration of 10% and pre-mixed biotin-labelled control oligo B2 and bioB, bioC, bioD and cre controls (Affymetrix cat# 900299) at a final concentration of 50 pM, 1.5 pM, 5 pM, 25 pM and 100 pM respectively. Targets were diluted in a hybridization buffer at a concentration of 50 ng/μl, denatured at 99°C for 5 minutes, incubated at 45°C for 5 minutes and centrifuged at maximum speed for 5 minute prior to introduction into the GeneChip cartridge. A single GeneChip® Human Genome U133A 2.0 was then hybridized with 1each biotin-labeled target. Hybridizations were performed for 16 hours at 45°C in a rotisserie oven.

GeneChip® cartridges were washed and stained in the Affymetrix Fluidics Station 450 following the FS450_0002 standard protocol. GeneChip arrays were scanned using an Affymetrix GeneChip® Scanner3000 7G. Affymetrix GeneChip® Command Console software (AGCC) was used to acquire GeneChip® images and generate.DAT and.CEL files, which were used for subsequent analysis with proprietary software.

Expression profiles, preprocessed with the MAS5 algorithm, were exported to GeneSpring GX software version 7.3 (Agilent Technologies). According to the GeneSpring normalization procedure, in each analysis the 50th percentile of all measurements was used as a positive control within each hybridization array and each measurement for each gene was divided by the value corresponding to the control. The bottom 10th percentile was used for background subtraction. Among different hybridization arrays, each gene was divided by the median of its measurements in all samples. Data was then log transformed for subsequent analysis. Expression data was prefiltered by considering both MAS5 ‘Absolute Call’ flags and average expression measurements within each group analyzed. We selected probe sets called present or marginal (P or M) at least once across all samples. The prefiltering method removed those probe sets whose expression signal was constantly too close to the background throughout the entire set of samples. In order to find genes whose expression levels significantly differed between OM cells and fibroblast, we adopted a supervised method of analysis, using the GeneSpring software. Mean values were calculated within the two classes for each probe set, and fold-change ratios between the OM and the fibroblast were derived. A difference of twofold cutoff was applied to select upregulated and downregulated genes. A further statistical analysis was performed using Welch’s approximate t-test and ANOVA, with P-value cutoff of 0.05, without the assumption of equality of variances. Benjamini and Hochberg false discovery rate (FDR) was used for multiple testing. By this analysis, 5759 probe sets were found to be significantly regulated between the two classes of samples.

## Results

### Cell populations in the olfactory epithelium tissue

Biopsies of the nasal OM were taken from the superior turbinate and the posterosuperior part of the septum, both areas are located in the upper nasal cavity, close to the cribriform plate. The specimens collected were used for the characterization of cell populations within the OE [9, 10]. Table 1 shows patients characteristics.

**Fig 1:**
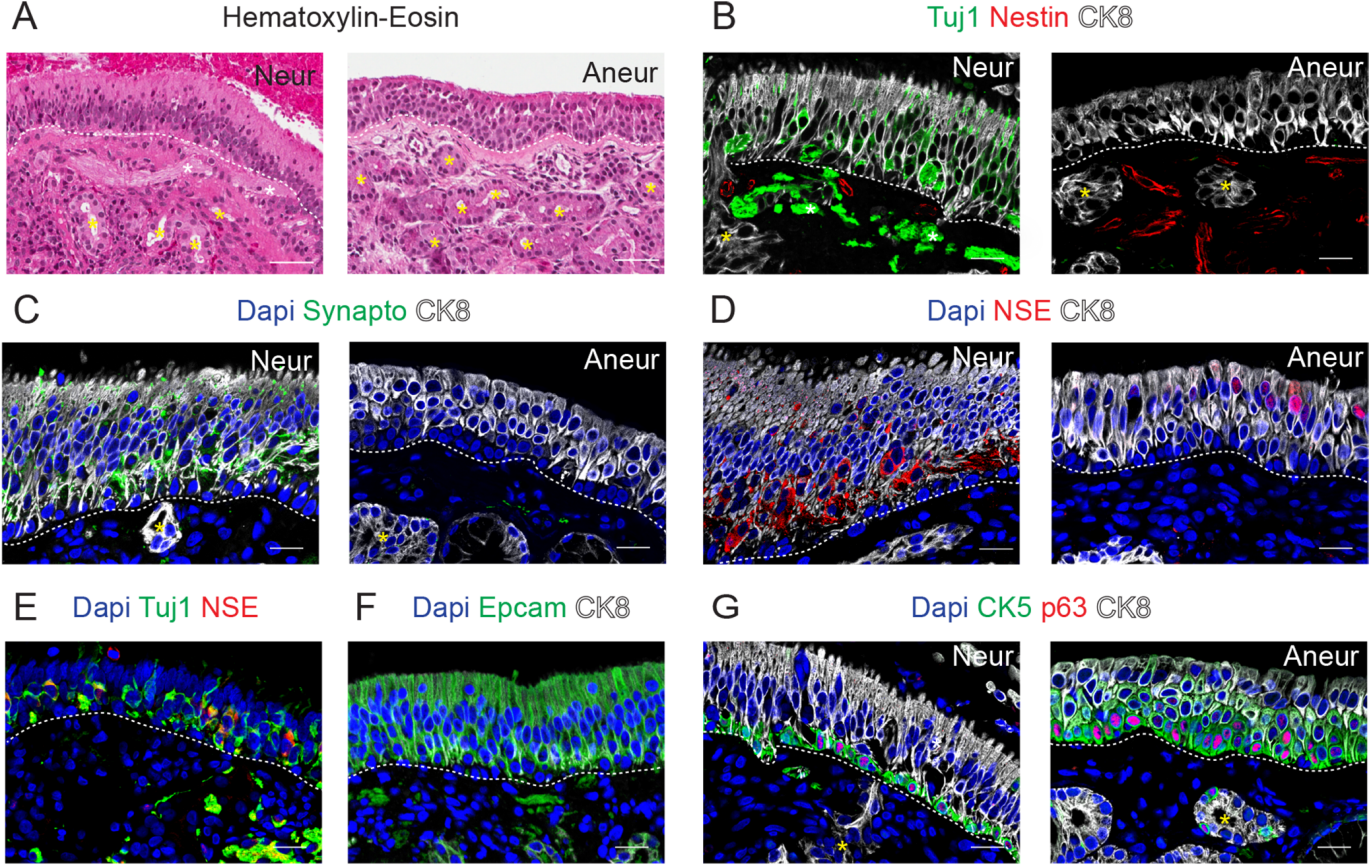
Characterization of the human olfactory epithelium. Hematoxylin-Eosin staining of the neuronal and aneuronal (Neur, Aneur) samples in A and immunostaining for Tuj1 and Nestin in B, for Synaptophysin (Synapto) in C, for NSE in D, for Tuj1 and NSE in E, for Epcam in F and for CK5 and p63 in G. In most of the panels, CK8 immunostaining was performed and Dapi was used as nuclear counterstaining. Dashed lines indicate basement membrane, yellow asterisks indicate Bowman´s glands and white asterisk indicates axon fibers. Scale bar in A, 100 μm; in B-G, 20 μm.

After processing the samples, the quality and the cytoarchitecture of the histological sections were verified using hematoxylin-eosin staining (Fig 1A). Furthermore, immunostaining of the samples was performed in order to detect specific markers for cell lineages.

Analysis of the immunostaining for the neuro-specific marker Beta 3 Tubulin (Tuj1) showed that half of the patients presented Tuj1 positive cells characterized by a bipolar spindle shape (n = 4; Neur sample in Fig 1B). Tuj1 labels the cytoplasm of the olfactory neurons and is found in the soma, the dendrites (that extend from the soma to the surface of the OM for the detection of the odorants) and the axon (that crosses the basal lamina and links to other processes forming bundles of unmyelinated olfactory fibers that reach the olfactory bulb) (Neur, Fig 1B). The absence of Tuj1 in some biopsies indicates that an aneuronal epithelium can be found at least in some areas of the OE (Aneur sample, Fig 1B). Indeed, analysis of two samples collected from the same patient (number 7) revealed that Tuj1 positive cells were found only in one of the biopsies, confirming the observations reported in previous studies that describe patches of non-neuronal tissue in the OE [10–12].

A similar pattern of distribution was obtained with immunostaining for Synaptophysin, which is a synaptic vesicle protein (Fig 1C) [13].

However, when OE was labeled with an antibody against Neuron-specific Enolase (NSE), which is a marker of metabolically active neurons [14], all the biopsies displayed NSE positive cells (Fig 1D). In the Neur samples, in which Tuj1 and Synaptophysin positive cells were present, most of the cytoplasmic expression of NSE colocalized with Tuj1 and Synaptophysin in both the OE and the nerve bundles (Fig 1E and not shown). Interestingly, in those samples that resulted to be negative for Tuj1, NSE was found to be mainly distributed in the apical layers of the OM, where sustentacular cells are typically placed, indicating that NSE is not exclusively a neuronal marker. A similar expression pattern was found in the mid turbinate where only a few Tuj1 positive cells were found (data not shown). In fact, albeit NSE is widely used and accepted as a neuronal marker, it has been shown to be expressed in other cell types such as glial cells and oligodendrocytes [15, 16].

In addition to the olfactory sensory neurons, the OE contains sustentacular cells and basal cells. Sustencular cells express epithelial markers such as CK8 and Epcam (Epithelial cell adhesion molecule) in the broad supranuclear fraction, that includes the apical surface lined by numerous microvilli and in the thin portion below the nucleus, that is attached to the basal lamina through foot processes (Fig 1B and 1F). Accordingly, sustentacular cells have been previously described to express CK18, which is a marker of epithelial luminal cells in rodents and humans [10, 17].

Horizontal basal cells are located within the basal lamina and they are considered to be stem cells capable of generating neuronal and non-neuronal cells of the OE [18–20]. They share many morphologic and histochemical features with basal cells of the nasal respiratory epithelium, such as the expression of p63 in the nucleus and CK5 (Fig 1G). It is noteworthy that the thickness of CK5 expressing cells was higher in Aneur biopsies, where no olfactory sensory neurons were found (Fig 1G). In addition, markers distribution was gradually changing since CK5 p63 cells were located in the basal layer, some p63 CK5 CK8 triple positive cells were found just above the basal layer, whereas CK8 CK5 cells or only CK8 cells had been found in the apical layers, suggesting that intermediate cell types could also be present in the aneuronal OE. CK14 positive cells has been shown to be expressed in horizontal basal cells of rodents [18, 21] and is usually co-expressed with CK5 in the basal epithelial cells [22]. Interestingly, CK14 positive cells were rarely found in the OE (data not shown). Indeed, Holbrook et al. [10] did not find CK14 positive cells in humans, probably indicating that CK14 expression is differently regulated in diverse species. CK8 was not detected in horizontal basal cells; conversely we could observe that sustentacular cells were anchored in the basal lamina through thin cellular prolongations that expressed CK8 (Fig 1B and 1C).

Globose basal cells are located above the horizontal basal cells. Their identification is more complex, as described in both humans and rodents [10, 21]. They could be identified by an exclusion criteria, since they do not express either markers for horizontal basal cells (such as CK5 or p63) or markers of olfactory neurons (such as Tuj1). It has also been described that globose basal cells are negative for CK18 [10, 17], therefore we expect these cells to be negative also for CK8. Indeed, we observed that some cells above the horizontal basal monolayer did not express CK8.

### Characterization of the lamina propria

**Fig 2:**
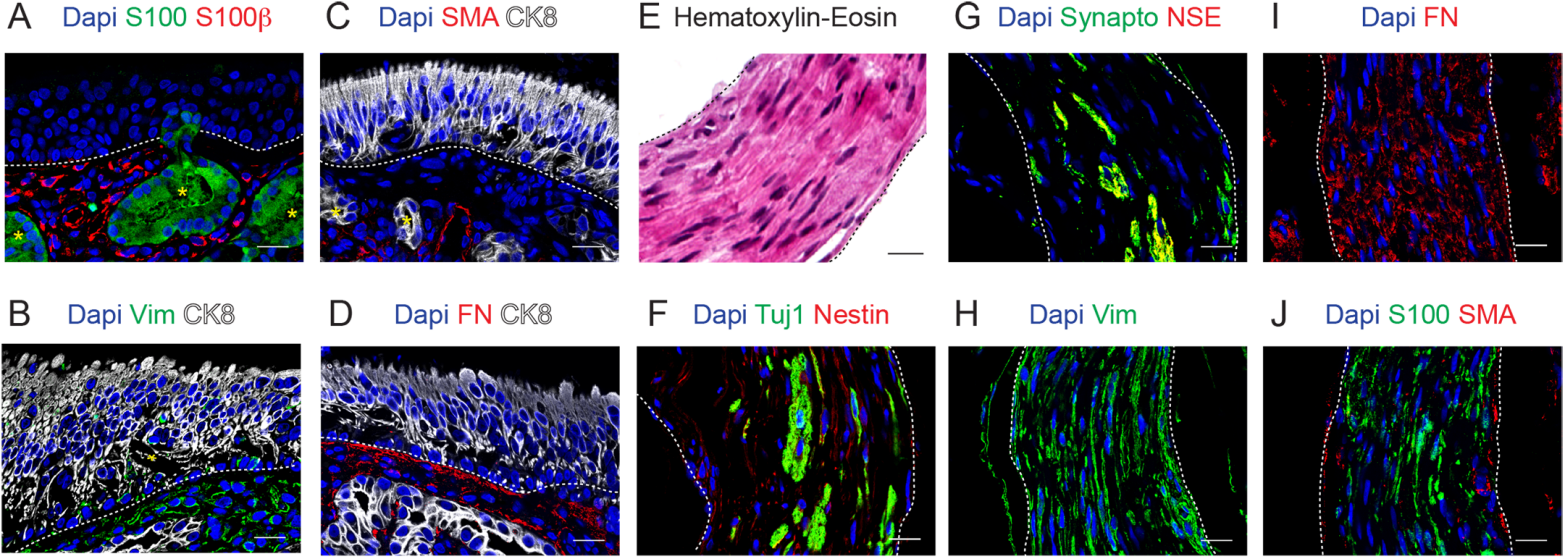
Characterization of the olfactory lamina propria. Immunostaining of the olfactory mucosa for S100 and S100β in A, Vimentin (Vim) and CK8 in B, SMA and CK8 in C, Fibronectin (FN) and CK8 in D. Hematoxylin-Eosin staining of nerve fascicles (E) and immunostaining for Tuj1 and Nestin in F, for Synapto and NSE in G, for Vimentin (Vim) in H, for Fibronectin (FN) in I and for S100 and S100β in J. In all the immunofluorescence panels, Dapi was used as nuclear counterstaining. In OM, dashed lines indicate basement membrane and yellow asterisks indicate Bowman´s glands. In E-J, dashed lines indicate the limits of nerve fascicles. Scale bar, 20 μm.

The lamina propria is located just beneath the epithelium and is composed of loose connective tissue and olfactory Bowman´s glands. The mucus secreted by these tubuloalveolar glands is carried towards the surface of the OE by narrow ducts passing through the epithelium (Fig 2A). Cells located in both the compact acini and ducts expressed CK8; and a few CK8-negative cells located in the external layer of the Bowman´s glands expressed both CK5 and p63 (Fig 1G).Both the compact acini and ducts express CK8 and very few CK5 and p63 double positive cells, negative for CK8, can be found in the external layer of the Bowman´s glands (Fig 1G). The calcium binding protein S100, which is a general marker for glia [23], was shown to be expressed in the glands but not in the OE, as previously reported (Fig 2A, [24]). The connective tissue presented immunoreactivity almost exclusively for Nestin, S100β, SMA, Vimentin and Fibronectin (Figs 1B and 2A–2D). Interestingly, some Vimentin positive cells were found to be immersed in the olfactory epithelium (Fig 2B). The presence of mesenchymal cell markers in the OE has been previously reported [5, 6]. One OM sample presented nerve fascicles, some of the cells were neuronal (ie. positive for Tuj1, Synaptophysin and NSE) and most of them expressed Nestin, Vimentin, Fibronectin, S100 and SMA (Fig 2E–2J).

### Proliferative activity in the OM

**Fig 3:**
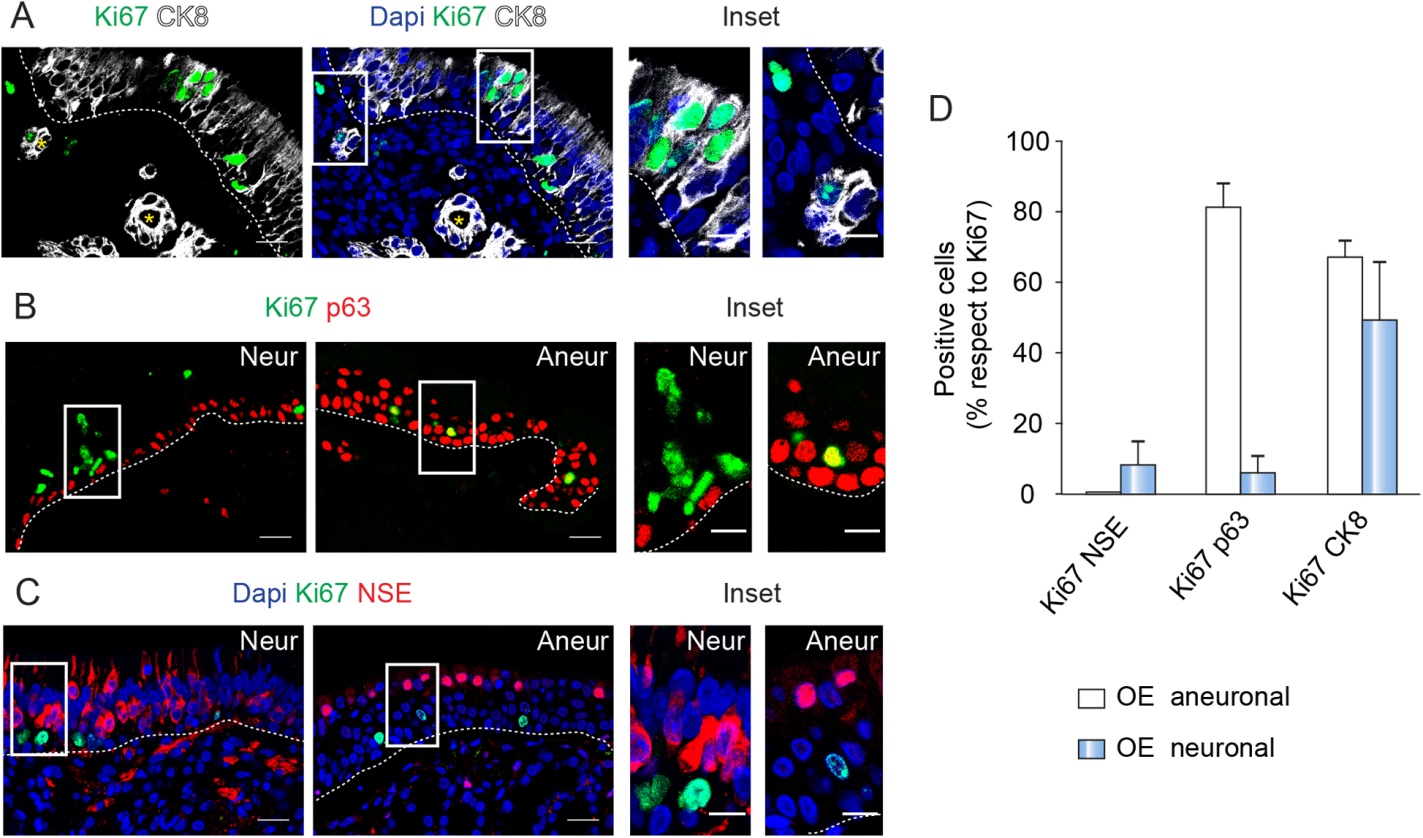
Different cell populations of the OM proliferates. In A, immunostaining of the OM for Ki67 and CK8. On the right, same area with Dapi staining. The insets enlarge positive cells in the OE and the lamina propria. Staining of the Neur and Aneur biopsies for Ki67 and p63 in B and for Ki67, NSE and Dapi in C. Positive cells are magnified in the insets. In A-C, dashed lines indicate basement membrane and yellow asterisks indicate Bowman´s glands. Scale bar: 20 μm and 10 μm in the inset. D, Quantification of double positive cells Ki67 NSE, Ki67 p63, Ki67 CK8 respect to total Ki67 cells in neuronal and aneuronal OE. Data are presented as mean ± SEM (n = 3–2).

Ki67 is a nuclear protein expressed in all cell cycle phases except during the resting phase and beginning of the G1 phase. Immunostaining for Ki67 was performed to address the proliferative activity of the OM [25]. Sparse Ki67-labeled nuclei were found outside the OE and a few Ki67 cells were present in the outer part of the subepithelial glands and in the connective tissue (Fig 3A). In the OE, some of the proliferating cells were CK8-positive sustentacular cells (Fig 3A). Other Ki67-positive cells, often forming small clusters, were located close to the basal layer. In neuronal samples, most of them did not express p63, suggesting that they could be classified as globose basal cells (Fig 3B). In contrast, the coexpression of Ki67 and p63 was frequent in aneuronal biopsies, confirming the presence of intermediate cell types in these samples (Fig 3B). Ki67 very rarely colocalized with NSE in either neuronal or aneuronal sections (Fig 3C). All this data indicated that some cell populations of the OM, such as globose basal cells, sustentacular cells and cells from the lamina propria, are highly proliferative (see all quantitative data in Fig 3D).

### Olfactory mucosa cells express ectomesenchymal markers in culture

Biopsies from human nasal mucosa were enzymatically disaggregated and the single-cell suspensions obtained were cultivated with a successful culture establishment rate of 6:6 samples.

With the objective of determining the phenotypic identity of the nasal cells in culture, we performed several antigen-based assays to assess the expression of different cell-specific antigens among our cultures.

**Fig 4:**
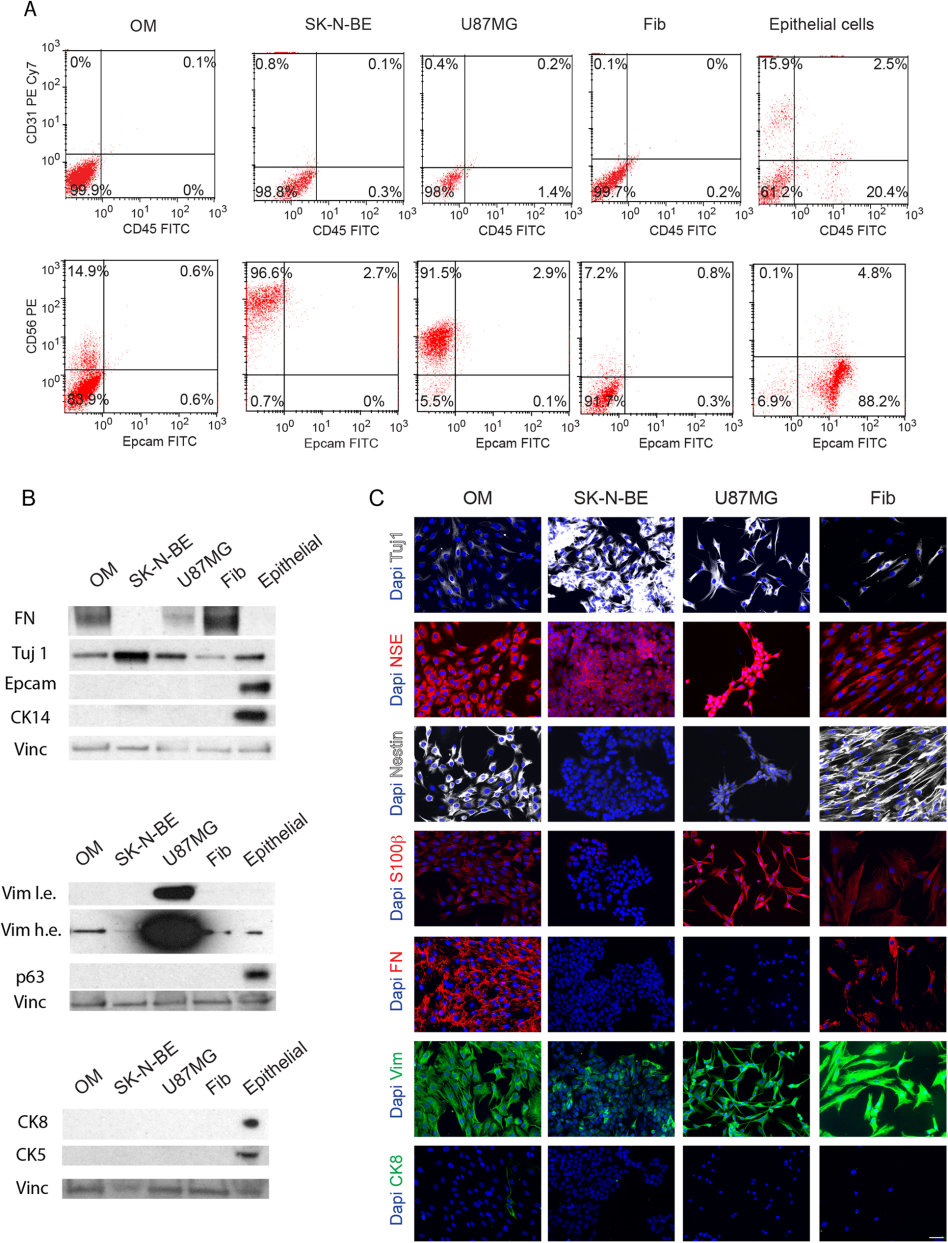
Antigenic markers of OM cells in culture. A. Representative FACS data of OM cells, SK-N-BE neuroblastoma cell line, U87MG glioblastoma cell line, primary fibroblasts (Fib) and primary epithelial cells. The antibodies used were anti-CD31 PE Cy7 and CD45 FITC in upper panels, and CD56 PE and Epcam FITC in lower panels. Data are presented as dot plots to visualize the expression of each marker. The percentage of the gated population is included. B. Western blots to detect Fibronectin (FN), Tuj1, Epcam, CK14, Vimentin (Vim l.e. and h.e., low and high exposure), p63, CK8 and CK5. Vinculin (Vinc) was used as loading control. C. Immunostaining for Tuj1, NSE, Nestin, S100β, FN, Vim and CK8. Dapi was used to stain nuclei. Bar, 50 μm.

To perform these assays, we used other cell cultures as references and controls: (1) SK-N-BE neuroblastoma cell line, (2) U87MG glioblastoma cell line, (3) primary fibroblasts and (4) primary epithelial cells. Our cultures were first subject to FACS analysis for anti-CD31 (also named, PECAM-1 or platelet endothelial cell adhesion molecule) and anti-CD45, that represent markers for endothelial cells and hematopoietic cells, respectively (Fig 4A). None of the olfactory cells in culture expressed CD31 or CD45, although positive cells were found among epithelial cells (Fig 4A), indicating that we were not isolating cells of endothelial or hematopoietic nature. We also observed by FACS and western blot that Epcam was not expressed in our cultures (Fig 4A and 4B). In addition, the presence of epithelial basal markers (CK5, CK14, p63) was not detected by western blot or immunofluorescence and only a few CK8-positive cells were found in the adhesion culture (Fig 4B and 4C and data not shown). These results suggested that we were not maintaining in culture epithelial nasal cells such as sustentacular cells, horizontal basal cells or cells from Bowman´s glands. GFAP expression was not detected in culture, indicating that we were not isolating unsheathed olfactory cells (not shown).

In contrast, FACS analysis evidenced a considerable proportion of cells that were positive for anti-CD56 or NCAM (neural cell adhesion molecule), whereas other cells were labeled with Tuj1 or NSE antibodies, indicating its ectodermal origin (Fig 4A–4C). Most of the cells were also positive for Nestin, S100β, Fibronectin and Vimentin (Fig 4B and 4C).

It should be noted that a similar immunoreactivity was detected in primary fibroblasts cultivated under the same conditions suggesting, in accordance with previous data, a low specificity of these antigens to distinguish between OM cells and fibroblasts obtained from human lung or breast primary samples [26].

### Identity of OM cells

The fact that OM derived cell cultures expressed antigens that were also present in primary fibroblasts led to a more detailed analysis of OM-derived cell cultures.

**Fig 5:**
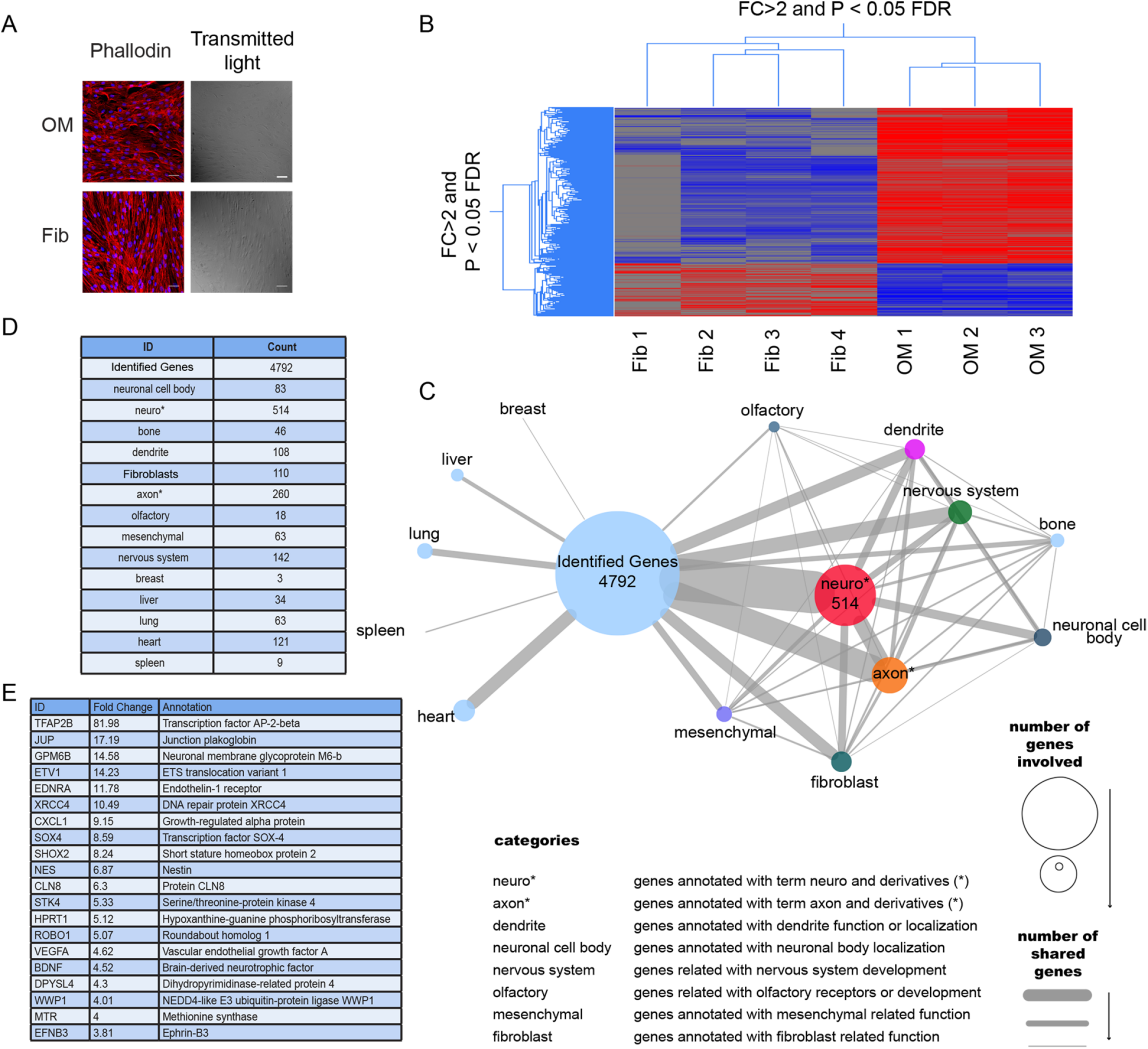
Identity of OM cells in culture. A. Actin staining using phalloidin and nuclei staining using Dapi of OM cells and fibroblasts. Images taken in bright field are also presented. B. Hierarchical clustering of transcript expression (> 2 fold, p <0.05) of OM derived cells was compared to already published fibroblast transcriptome data. C. A network-based organization of the relationship genes-GO terms for the 4972 genes identified in our cells is shown; nodes represent number of genes that share a term related to nervous system function and/or localization (i.e. axon, dendrite, neuronal cell body etc.) in comparison with other organs function and/or localization. Edges connecting nodes represent the number of genes sharing the same term. Node and edge sizes represent the number of genes possessing a determined term. D. Number of genes that among their GO annotations contain the displayed terms and derivatives of those terms. E. List of the top 20 up-regulated genes (>2 fold) that contains the term nervous system as GO annotation.

Our microscope analysis showed that OM cells differed in morphology from fibroblasts when grown in a culture; the aspect ratio of the nuclei was significantly different (1.52±0.05% *vs*. 1.90±0.10% respectively, n = 3, p<0.05; Fig 5A), suggesting a distinct identity of these cultures.

Thus, to better identify the nature of our cultures, we performed a microarray Affymetrix analysis using three independent OM cell preparations (Fig 5B). Transcript expression of OM-derived cells was compared to fibroblast transcriptome data available in published literature (from which we only extracted the profile of Synovial Fibroblast (SF) cells (GSM606428, GSM606429, GSM606430, GSM606431). (accession number GSE24598)) [5].

To determine whether OM cells and fibroblast cells were molecularly distinct, we carried out a supervised analysis to select genes whose expression levels were significantly different between the two cell types (≥ 2.0-fold change, P-value <0.05 with multiple testing correction). This analysis identified a signature of 5759 probe sets, corresponding to 4972 unique genes (S1 Table). By performing a hierarchical clustering we observed that the two populations showed a clearly different expression profile, although all the 3 OM-derived cultures displayed an homogeneous transcriptome.

Interestingly, although we observed that fibroblasts and OM-derived cultures shared the expression of several antigens, the transcriptional pattern between those cell types differed strongly.

We also identified the enriched Gene Ontology terms (cellular component and biological processes) for the unique 4972 genes identified in our cells and then, established the level of relationship between these genes and relevant GO terms regarding organ/system functions, cellular types and cell type localization; we were then successful in organizing them in a network (Fig 5C and 5D).

As shown in Fig 5C and 5D, the highest level of association was established with GO terms containing the sub-term “neuro”, also with a high association between "nervous system", "axon" and "neuronal cell body" terms. In comparison, other terms related to other organ/systems had a lower level of association with the identified genes, indicating that the changing genes could potentially establish a neuronal state.

Fig 5E lists the top 20 up-regulated genes (>2 fold) expressed in OM respect to those expressed in fibroblasts that contains the term nervous system as GO annotation, showing that these genes are directly related with neurological functions.

### Stem cells purification from clonal spheres

**Fig 6:**
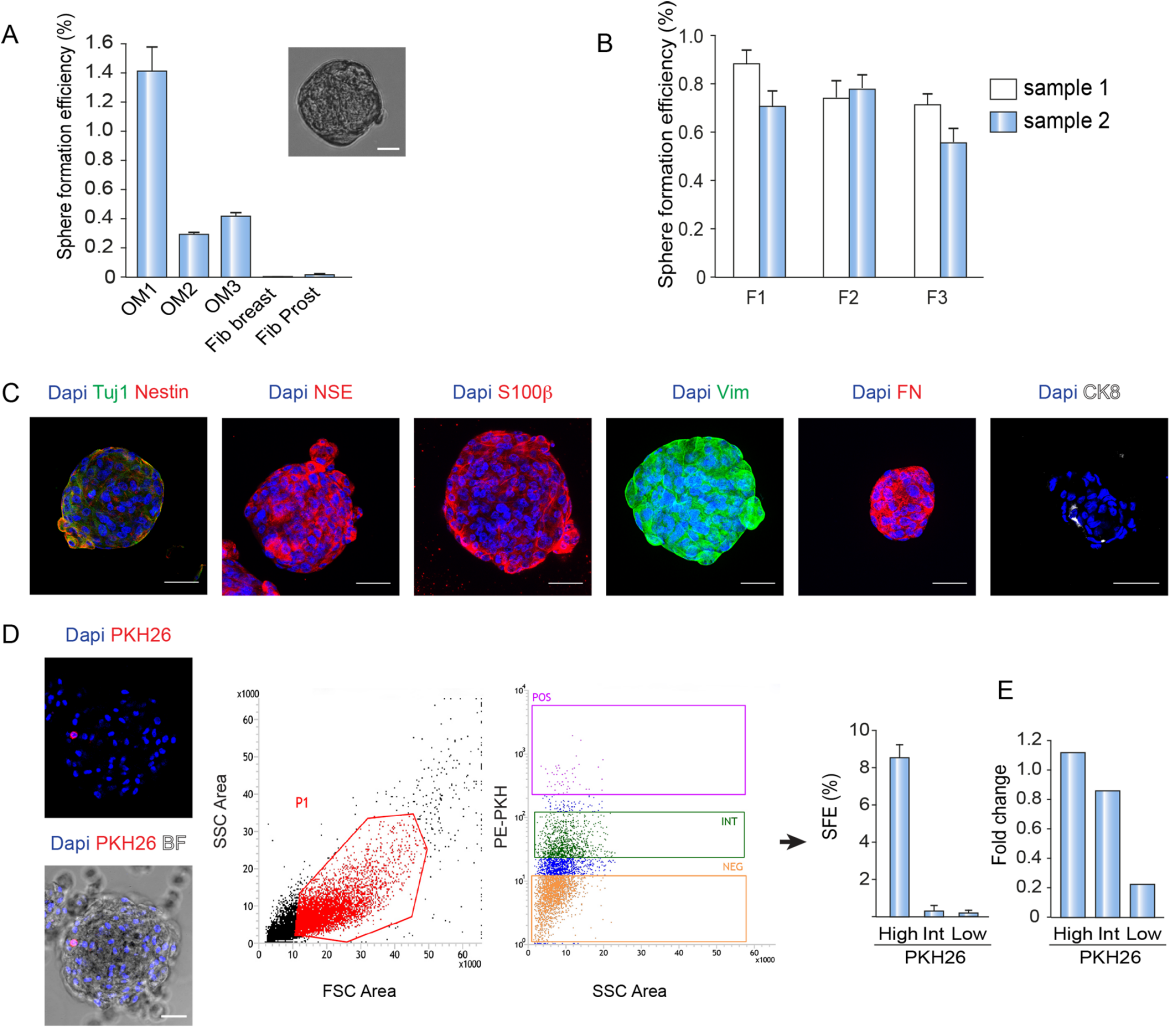
Characterization of OM spheres and method to isolate putative OM stem cells. A. SFE of OM samples and fibroblasts in the presence of methylcellulose. Example of a typical OM sphere. B. SFE of OM samples in suspension culture for 3 generations. Data are presented as mean ± SEM (n = 3). C. Immunostaining of OM spheres for Tuj1, Nestin, NSE, S100β, Vim, FN and CK8. Dapi was used to stain nuclei. D. On the left, example of an OM sphere labeled with PKH26 and Dapi. Representative FACS data of OM cells labeled with PKH26. On the right, sorted populations (positive, intermediate and negative) are shown. Quantification of SFE obtained from each sorted population. Data are presented as mean ± SEM (n = 3). E. Quantitative RTPCR for the expression of CD271 in PKH26-labeled cells. Data were normalized to the reference gene β*-ACTIN* (ACT). Bar, 50 μm.

We next determined to establish if our OM cultures contain cells with stem cells characteristics. We approached this question by assessing if our cell cultures could generate clonal three-dimensional spheres in suspension growth conditions, in particular in a serum-free medium supplemented with mitogens. To do so, disaggregated cells were seeded in poly-HEMA-coated plates (to avoid cellular adhesion to the plastic) and in the presence of 1% methylcellulose to prevent the formation of aggregates. Sphere formation efficiency (SFE) was considerably higher in OM cell cultures than in fibroblasts, demonstrating the presence of sphere initiating cells in the OM cultures and therefore suggesting the presence of stem cells within in vitro cultures of human OM (Fig 6A).

To better characterize these putative stem cells, we determined if the OM spheres could be serially passaged, as typically done by stem cells [8, 27]. Therefore, we generated a first sphere generation (called F1 generation) that was successively dissociated: the resulting suspension was re-plated to generate the next consecutive generation of spheres (F2). As shown in Fig 6B, OM spheres could be serially passaged for at least 3 times and presented a SFE that remarkably did not decrease substantially in the successive generations. When OM spheres were immunostained using different markers, we found that spheres stained positive for Tuj1, NSE, S100β, Vim and FN (Fig 6C). In agreement with the analysis performed on adhesion OM cultures, OM spheres did not express basal epithelial markers and only a few contained 1 or 2 CK8-positive cells per sphere (9±1.4% of the total spheres, n = 2; Fig 6C and data not shown).

Stem cells could perform one or two rounds of division and then return to a quiescence state. Considering the presence of putative stem cells in our cultures and with the intent of identifying and isolating them, we used the PKH26 technology [27–29]. The PKH26 staining allows the stem cells to be identified as a consequence of their ability to retain a lipophilic fluorescent dye (PKH26) [27–29], which is progressively diluted during proliferation in the precursor’ progeny.

We prepared a pool of 3 different OE-derived cultures that were stained with PKH26; then we put them in a suspension culture to allow the formation of a primary generation OM spheres. After 7 days, most of the cells that formed the OM-derived spheres were not stained by PKH26, confirming the expected dilution of the dye during cell proliferation while few cells were still PKH26-labelled (Fig 6D). Then, we trypsinized the spheres and re-plate the cells to allow the formation of a second generation of spheres to further dilute the dye.

Cells from the second generation of spheres were dissociated and different fractions of PKH26-labeled OM cells were purified by FACS sorting (Fig 6D) based on the intensity of the dye (bright, intermediate and negative). Afterwards, all the fractions were re-plated separately to generate further spheres in order to investigate the stem cell content in each fraction. Importantly, the SFE was higher in the PKH26-bright labeled cells than in the rest of the fractions, indicating that we were indeed enriching in OM stem cells (Fig 6D).

In previous studies, purification of stem cells located in the lamina propria was performed using a membrane marker called CD271, which is specifically expressed in mesenchymal stem cells [6]. In agreement with these results, we observed a higher expression level of CD271 in PKH26-positive labeled cells, indicating that PKH26 dye can be used to purify stem cells from human OM (Fig 6E).

## Discussion

In the current study we have taken advantage of the easy accessibility of the olfactory mucosa to perform non-invasive biopsies that were processed to characterize the cell populations contained in the OM. This complex tissue supports functional restoration after injury, suggesting that its turnover is driven by the differentiation of stem cells lying in the OM.

In agreement with previous findings, we observed that some of the biopsies lacked olfactory neurons [9, 10, 12]. Patches of neuronal disruptions in nasal epithelium could correspond to the OE margins [10], to zones where the olfactory neurons are depleted or, in the most extreme cases, to metaplastic areas of respiratory epithelium [9, 11, 12, 30]. It has been shown that neuronal discontinuity increases with age, but it is also influenced by specific conditions such as the presence of polyps [1, 11, 12]. Our data on the expression of p63 and cytokeratins suggests that the aneuronal epithelium is organized differently. We also observed that both Tuj1 and Synaptophysin could be used as markers to assess the presence of olfactory neurons, but this was not the case for NSE. Interestingly, the NSE distribution pattern was similar to that of the Protein Gene Product 9.5 (PGP9.5), which is another neuron-specific ubiquitin hydrolase, indicating that neither NSE nor PGP95 are good candidates to detect olfactory neurons [10].

In addition, our experiments indicate that different cell types are cycling in the OM. Some Ki67 positive cells were found in the connective tissue whereas others, such as some sustentacular and globose basal cells, were present within the OE. Further studies have also shown that the globose basal cells represent the highly proliferative precursor compartment in the OE, whereas the horizontal basal cells represent the quiescent stem cells [18–20].

Importantly, the cellular composition of the OM has been shown to be altered in neuropsychiatric and neurodevelopmental disorders, indicating that a detailed study of the OM could provide information on live patients, possibly leading to identification of illness biomarkers (revised in [31]).

In order to identify OM stem cells, we have successfully set up OM cell cultures from different human biopsies (6 out of 6 samples). We have been able to establish cell cultures even from samples that lacked olfactory neurons, indicating that the biopsies did not contain an homogeneous cell population, highlighting its intrinsic variability. Despite this heterogeneity, there was similarity among cell cultures when considering protein expression and transcriptional profiles.

Using specific antibodies, we have demonstrated that our cell cultures mostly expressed markers present in lamina propria. The expression of neuron-specific proteins (such as Tuj1 or NSE) was faint in comparison to neuroblastoma or glioblastoma cell lines and the presence of epithelial markers was very rare, suggesting that we did not isolate or maintain in vitro cells derived from the OE. Accordingly, these data indicates that our culture milieu was selecting cells belonging to the lamina propria, even though the OM tissue contained additional cell types. Therefore, we hypothesized that although other groups have shown that OM cultures can contain both non-epithelial and epithelial cells [6, 7], it might be more difficult to maintain epithelial cells in vitro, due to their slow rate of growth in a serum-free medium and to an impaired adherence to the plastic plate [7].

Albeit OM cells and fibroblasts displayed similar immunoreactivity, we observed that they had a different morphology and transcriptional pattern. Interestingly, further analysis on those genes that were differently regulated in these cultures demonstrated that most of them were related to the neuronal system. For example, among a panel of common secreted growth factors [32] we observed a high expression of the Brain-derived Neurotrophic Factor (BDNF), which is a protein secreted by both neural stem cells (NSCs) and mesenchymal stem cells (MSCs). Furthermore, our cells showed a high expression of Ephrin-B3 (EFNB3), which is a cell surface transmembrane ligand for Eph receptors, that induces synaptogenesis, modulates synaptic function and regulates spine morphogenesis [33]. In addition, a two-fold increase in Glycoprotein M6B (GPM6B) expression levels is also present in our cells. Glycoprotein M6B represents a neuroplasticity-related gene that interacts with the serotonin transporter and is down-regulated in the hippocampus of chronically stressed animals and in cases of depressed suicides [34, 35]. A further interesting example is Roundabout, Axon Guidance Receptor, Homolog 1 (ROBO1) whose expression is implicated in several tumors of the nervous system: a low expression of ROBO1 is related to brain metastasis indicating a poor prognosis, whereas a high expression is common in gliomas [36, 37].

In agreement with our results, Delorme et al [5] demonstrated that the transcriptomic profile of OM cells in culture was not closely related to primary fibroblasts of synovial origin. Interestingly, OM cells seemed to be highly similar to bone marrow stem cells, but with some singular characteristics like the over-expression of neuronal genes. Altogether these data indicates that OM cells could be considered as a subgroup of mesenchymal cells with neuronal traits. Their unique ectomesenchymal nature could be probably explained by their developmental origin, since they might arise from neural crest [38] or by a niche effect; indeed, they are found in a neuronal environment, in close proximity to a neurogenic area.

Several researchers have studied the multipotency of olfactory stem cells and it has been shown that they can generate many different cell types including neural cells, osteoblasts, adipocytes, chondrocytes and cochlear hair cells [6, 7, 39–41]. This potential for differentiation combined with a direct accessibility explains the interest in the cultivation and purification of cells from OM [6, 26].

Thus OM has been proposed as a source of stem cells for tissue regeneration. However, little is known about stem cell markers that may allow to purify them. Therefore, in order to identify and isolate OM stem cells we assessed the ability of OM ex-vivo cultures to form spheroids derived from a single cell in low attachment culture conditions. We have shown that OM derived cultivated cells are able to form free-floating spheres when seeded at low density in serum-free suspension cultures. Importantly, such spheres can be serially propagated suggesting the presence of stem like cells.

Unexpectedly we have observed that fibroblast cultures were also able to form spheres. However, it is noteworthy that the SFE was higher in cells derived from OM tissue than it was in terminally differentiated fibroblasts, confirming a high stem cell content in OM cells.

The immunostaining of the neurospheres have shown that the antigens expressed are similar to the OM adherent cells, suggesting that they originate from mesenchymal stem cells, as already reported [5–7].

Taking advantage of the quiescent nature of stem cells, we developed a method based on the functional labelling by PKH26 staining, with the intent of isolating and purifying putative stem cells. PKH26 has been previously used to isolate mammary stem cells from normal and tumoral breast tissue but this is the first paper that applies this technology to OM tissue [27–29]. By using PKH26, we were able to identify a fraction of cells that had a high content in stem cells, as shown by its high SFE value.

CD271 (Low-affinity nerve growth factor receptor) has been shown to be highly expressed in stem cells located in the lamina propria by immunostaining [6]. Lindsay et al [6] showed that these cells can form colonies and can differentiate (secrete fat and produce bone) by using a kit that select for CD271 expressing cells. Interestingly, CD271 was enriched in PKH26-positive cells by qPCR analysis. Although this marker could not clearly discriminate between the PKH26 high and intermediate cells, it could discriminate the negative-non forming spheres-fraction from the PKH-labeled cells, showing that either by PKH labeling or by CD271 staining, the cell population identified contains pluripotent cells. It will be interesting in a future work, to use both markers in combination to better characterize such populations.

Therefore, this work contributes to deepen the characterization of the human OM and importantly, it presents a method that can be used to enrich and isolate OM stem cells from an easy to access tissue. These stem cells could be differentiated in vitro and be beneficial to perform autologous transplantations or disease modeling [42, 43].

## Supporting information

S1 Table Genes differentially expressed in OM cells. List of the genes identified in OM cells whose expression levels significantly different in our analysis (≥ 2.0-fold change, P-value <0.05) together with their relevant GO terms regarding biological process, cellular localization and molecular function.
(XLSX)
